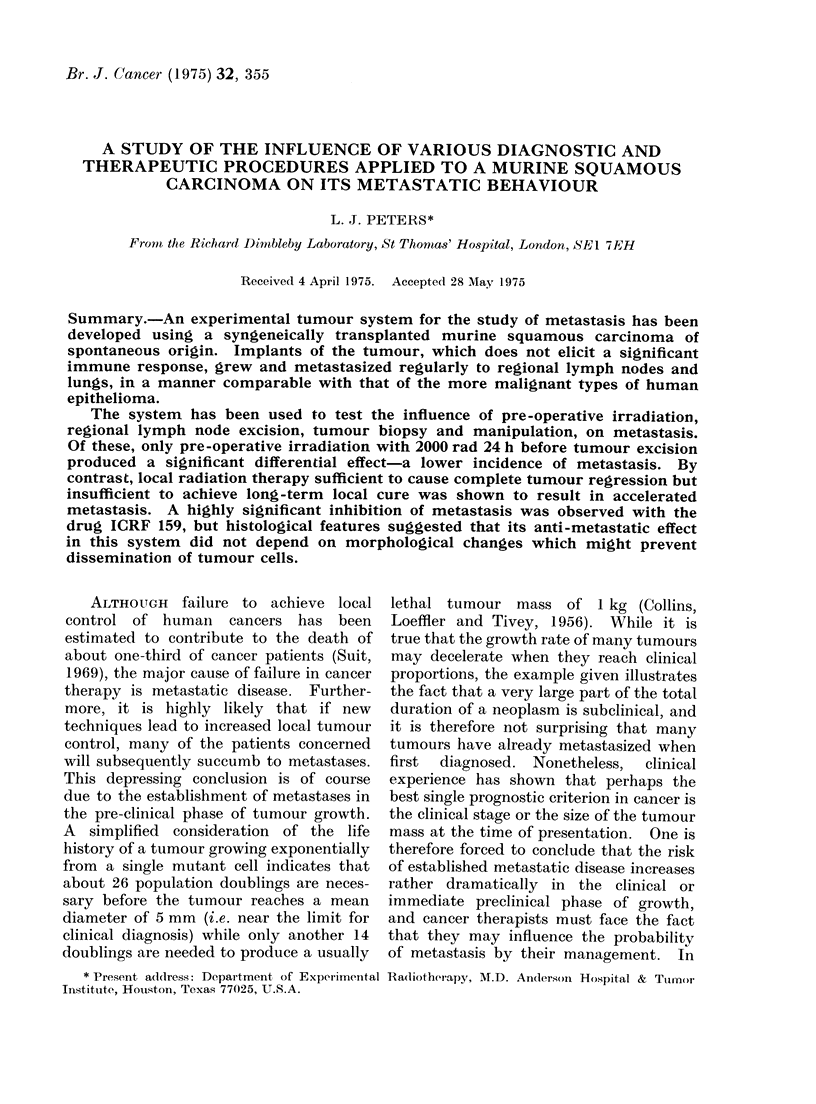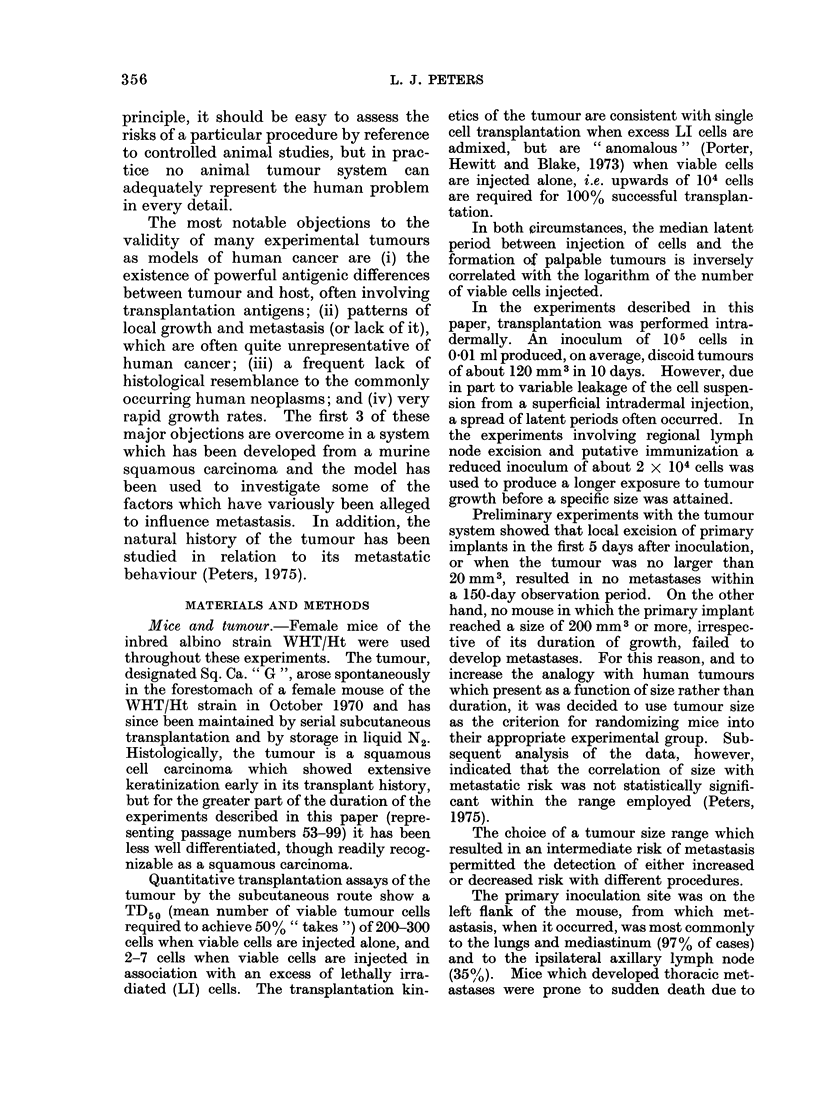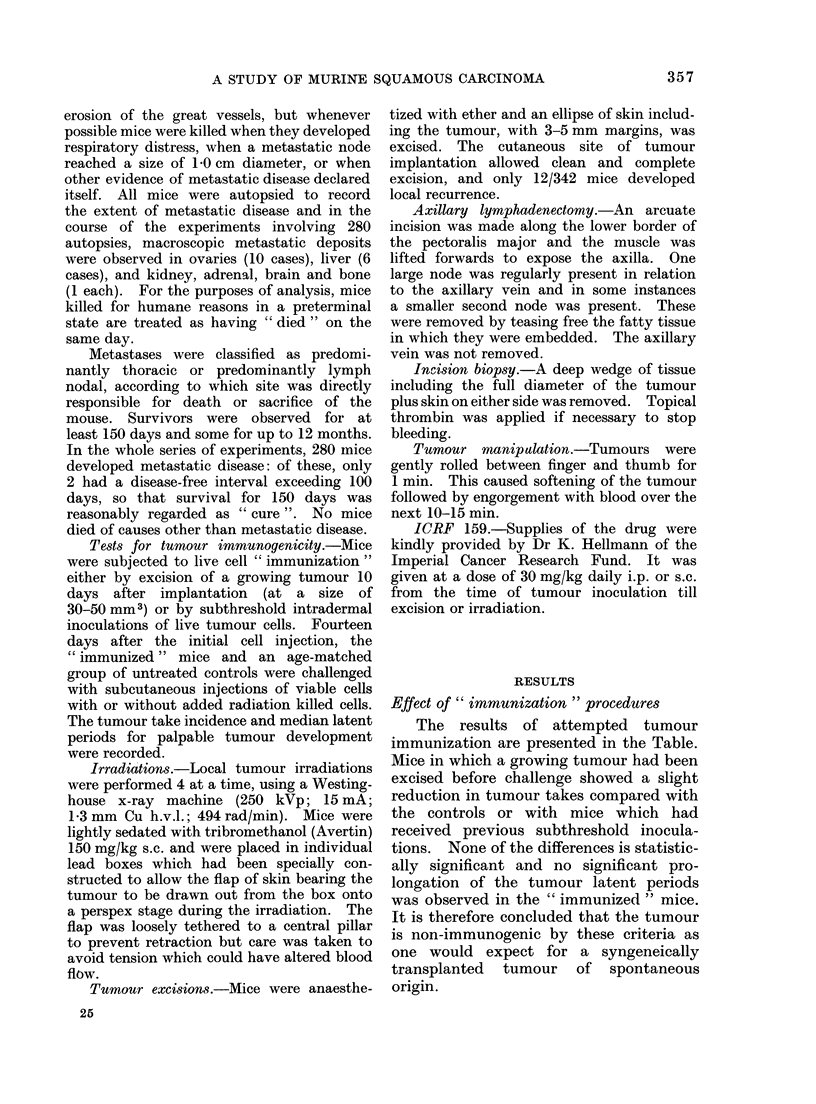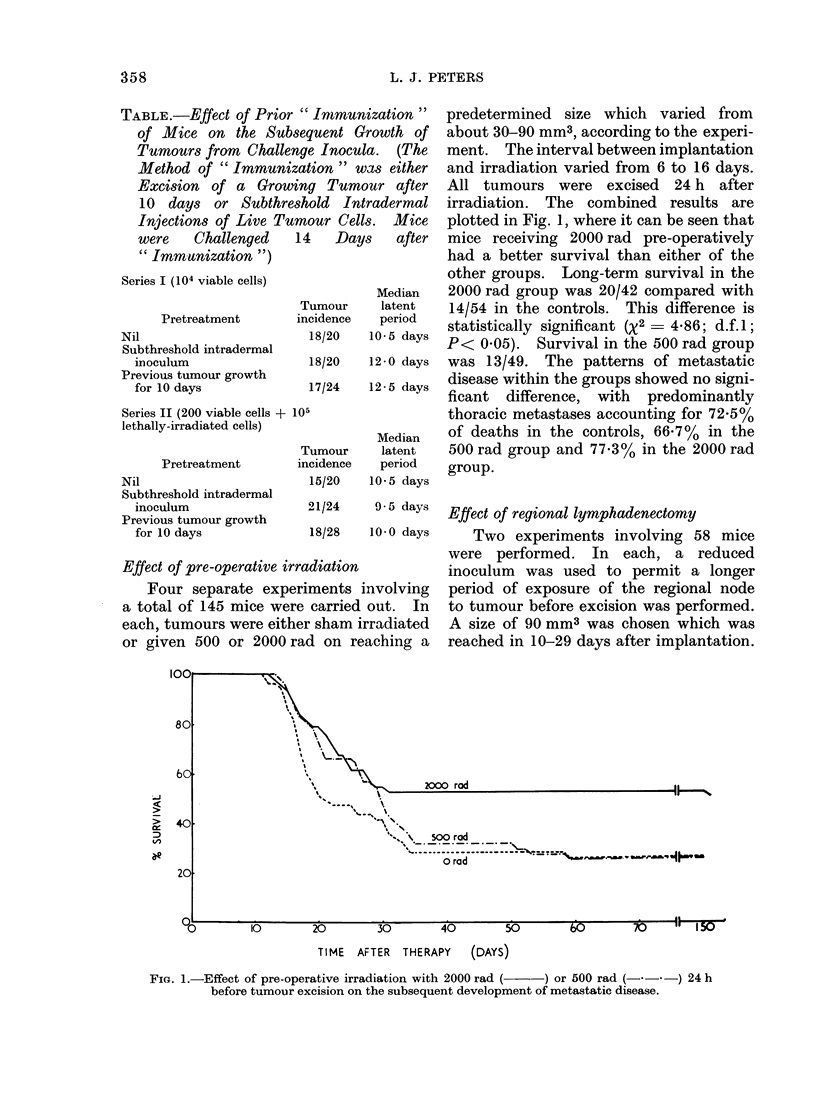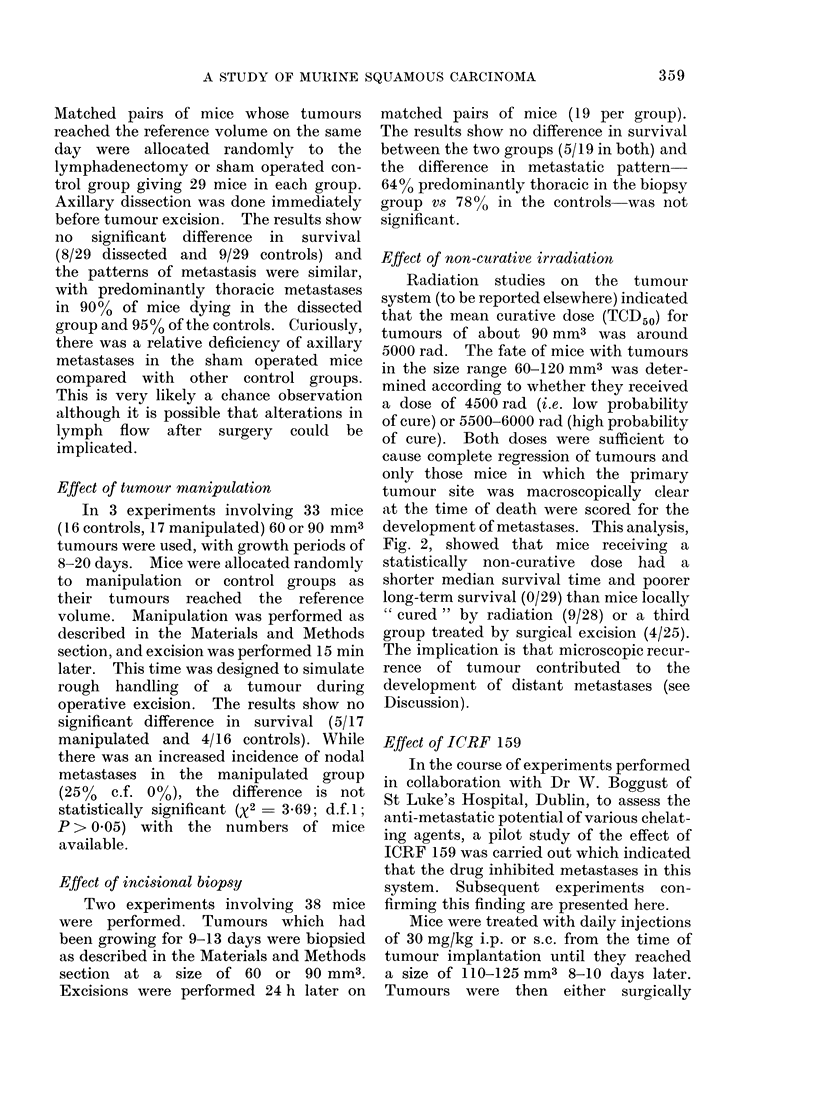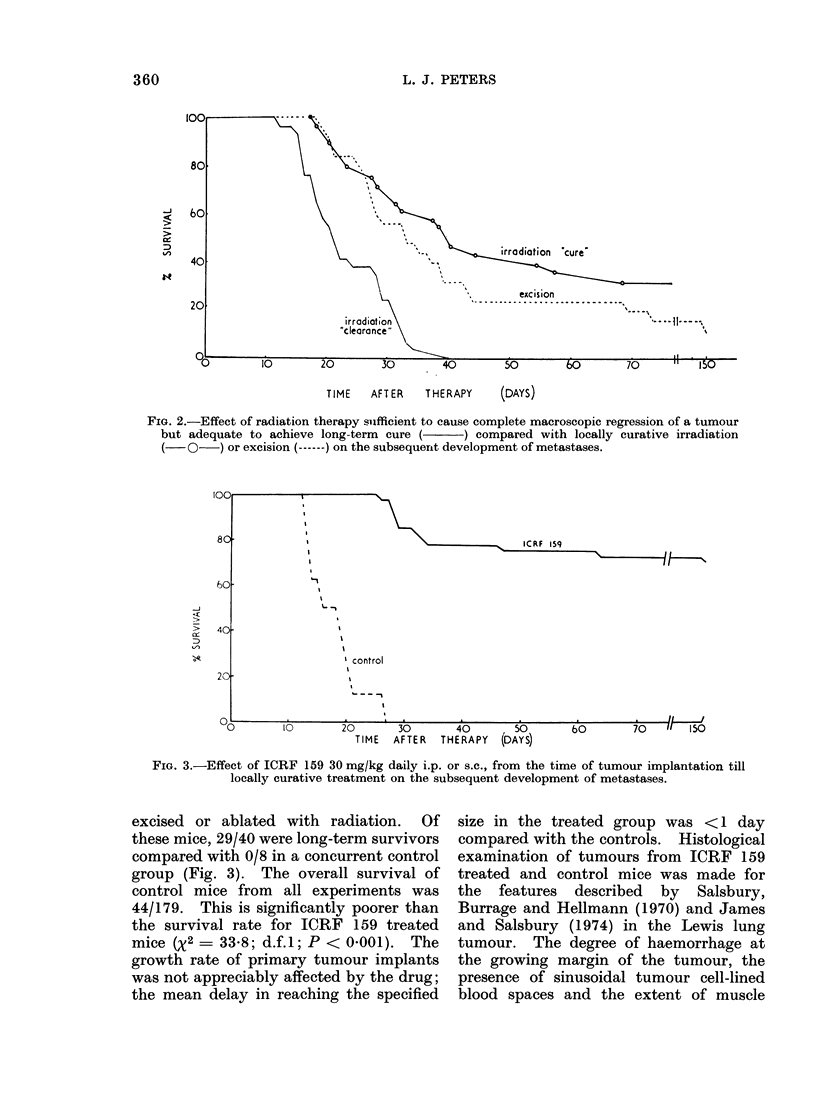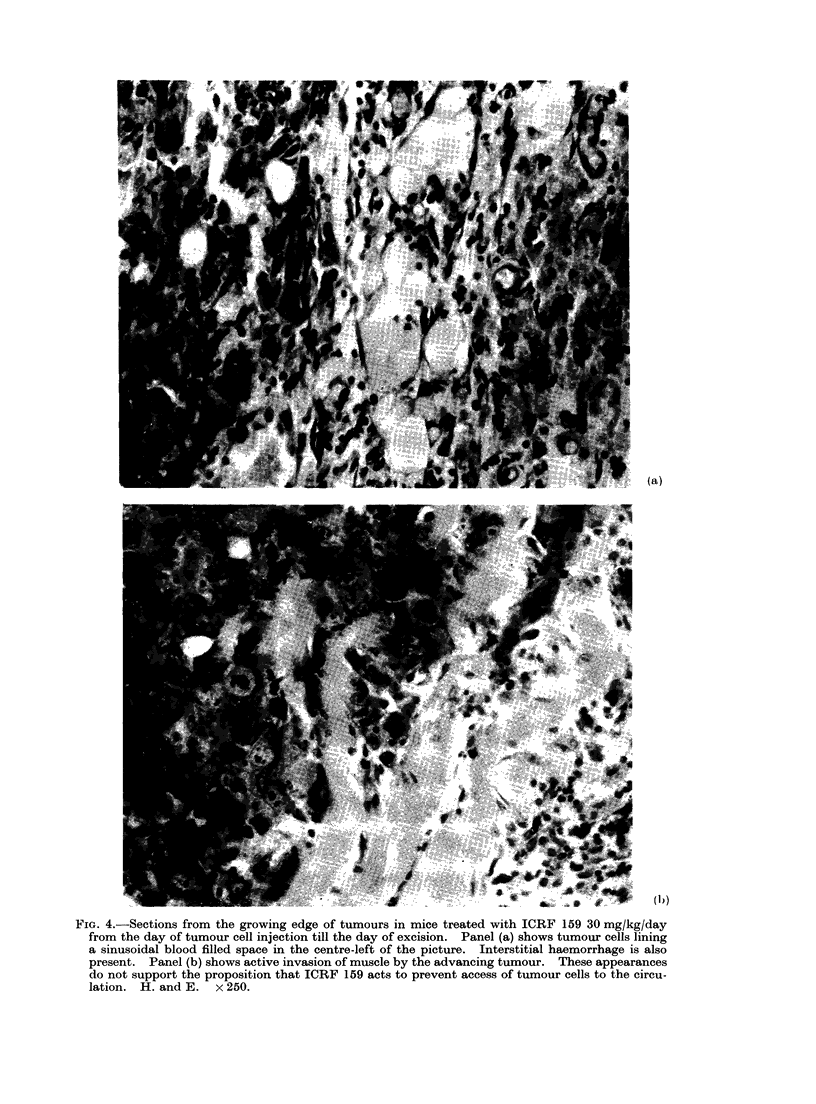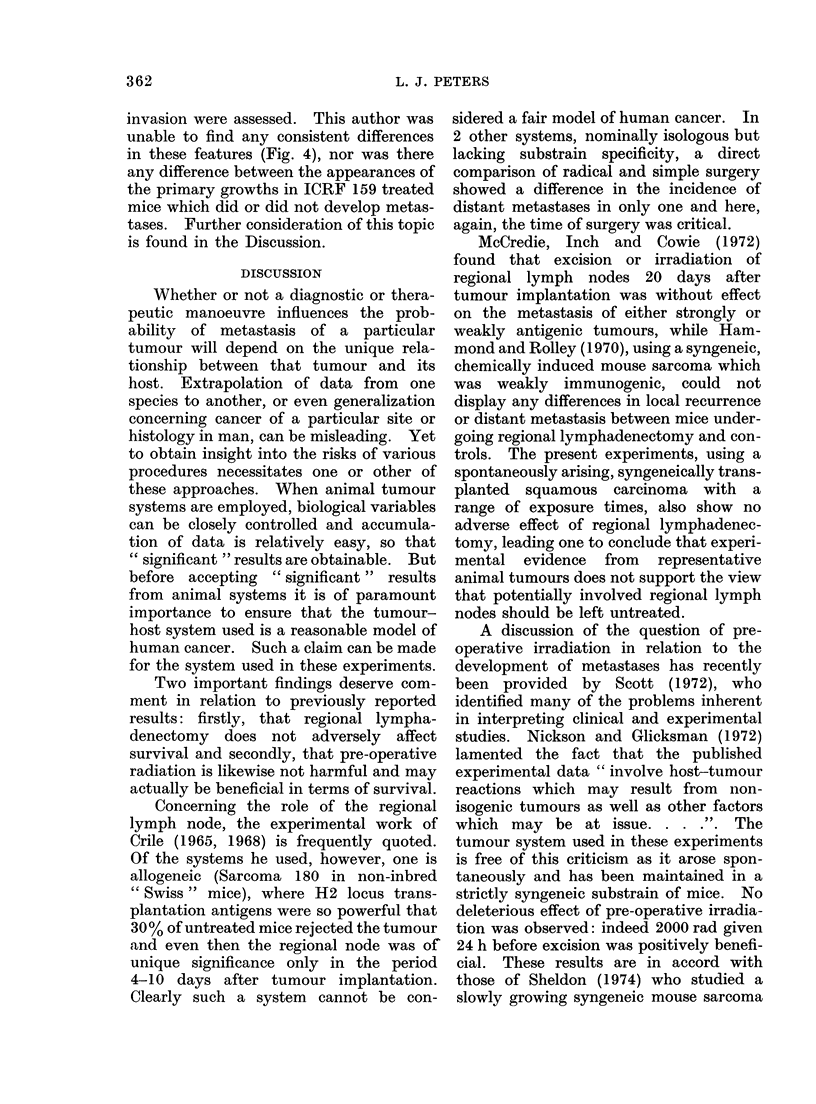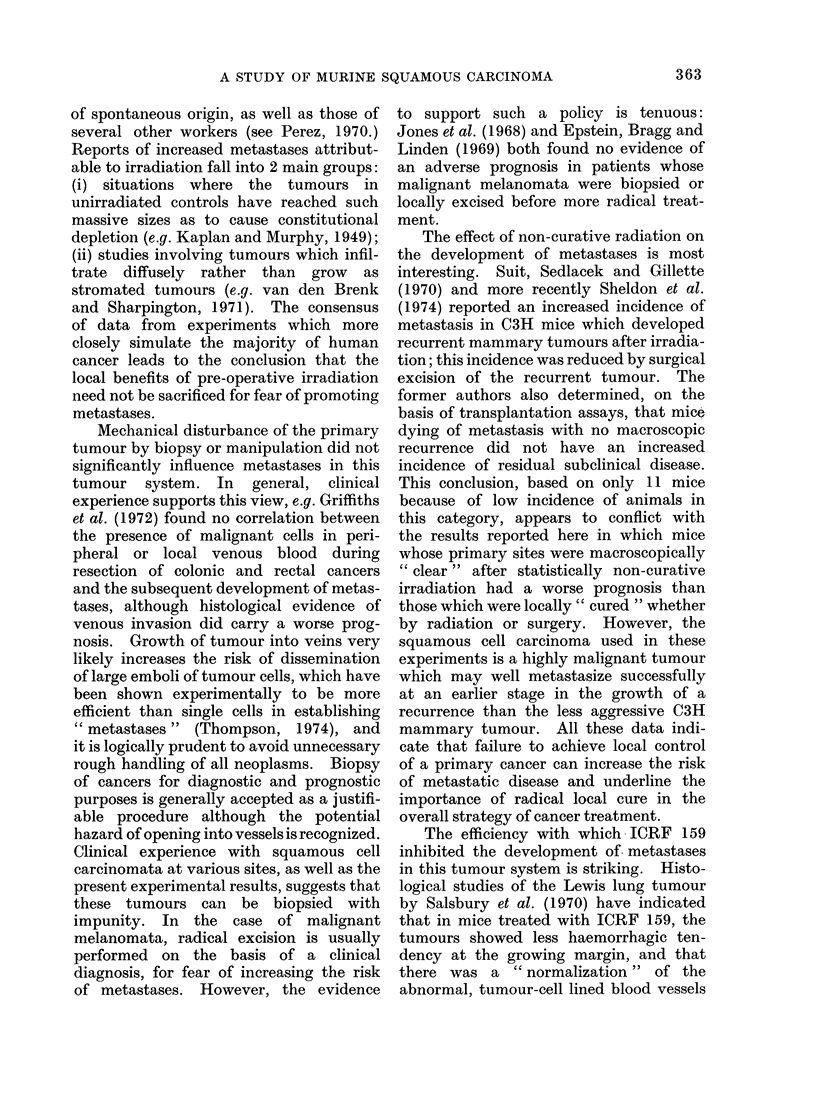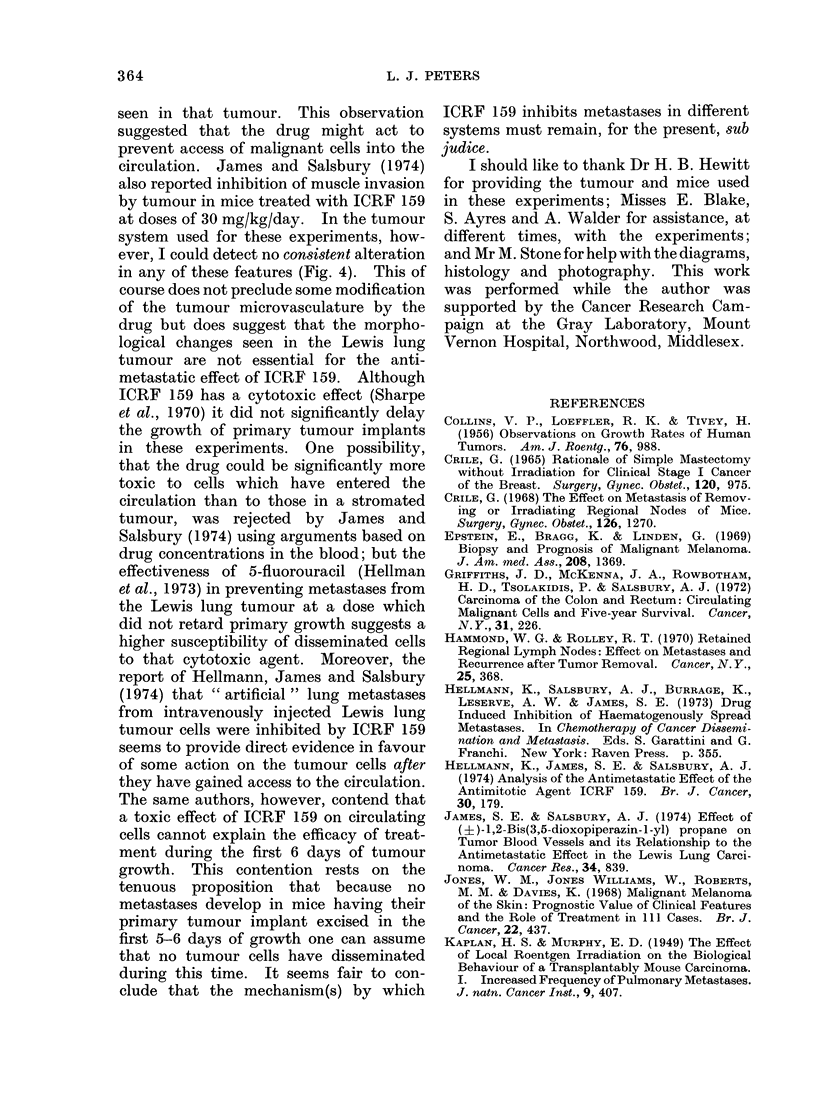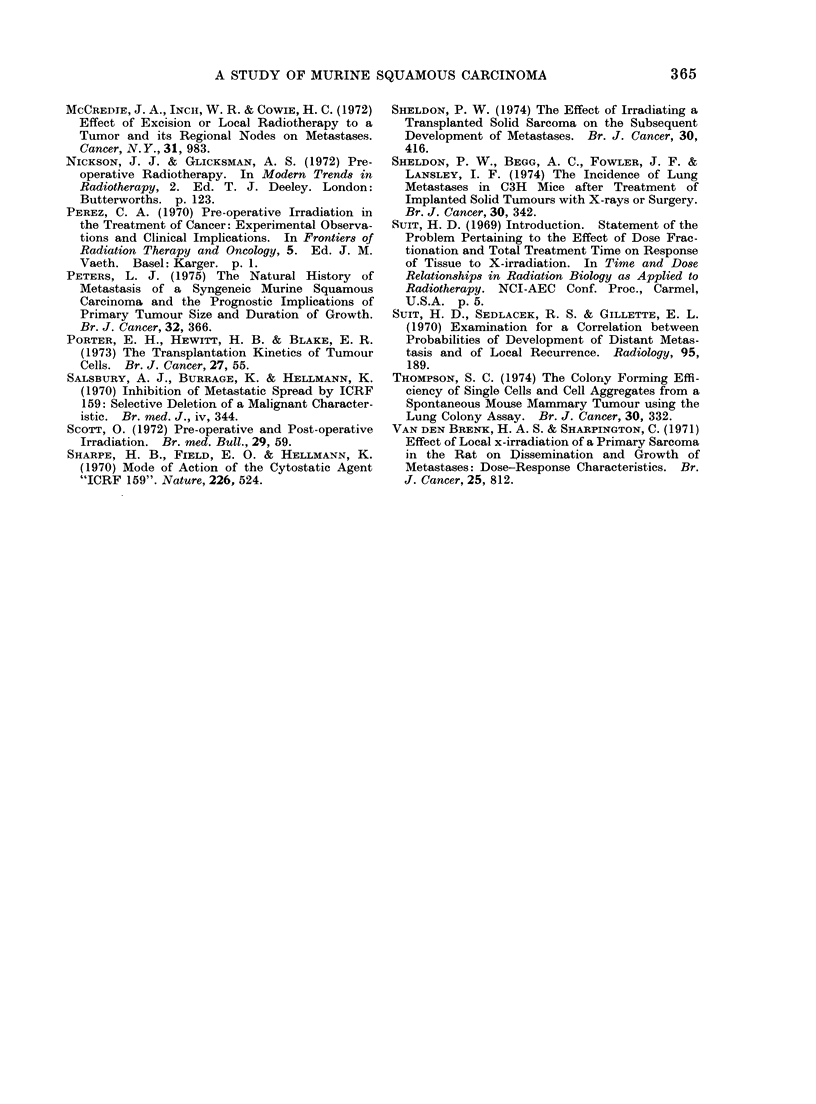# A study of the influence of various diagnostic and therapeutic procedures applied to a murine squamous carcinoma on its metastatic behaviour.

**DOI:** 10.1038/bjc.1975.235

**Published:** 1975-09

**Authors:** L. J. Peters

## Abstract

**Images:**


Br. J. Cancer (1975) 32, 355

### A STUDY OF THE INFLUENCE OF VARIOUS DIAGNOSTIC AND THERAPEUTIC PROCEDURES APPLIED TO A MURINE SQUAMOUS CARCINOMA ON ITS METASTATIC BEHAVIOUR

L. J. PETERS*

*From the Richard Dimbleby Laboratory, St Thomas' Hospital, London, SE1 7EH*

Received 4 April 1975. Accepted 28 May 1975

**Summary.—An experimental tumour system for the study of metastasis has been developed using a syngeneically transplanted murine squamous carcinoma of spontaneous origin. Implants of the tumour, which does not elicit a significant immune response, grew and metastasized regularly to regional lymph nodes and lungs, in a manner comparable with that of the more malignant types of human epithelioma.**

**The system has been used to test the influence of pre-operative irradiation, regional lymph node excision, tumour biopsy and manipulation, on metastasis. Of these, only pre-operative irradiation with 2000 rad 24 h before tumour excision produced a significant differential effect—a lower incidence of metastasis. By contrast, local radiation therapy sufficient to cause complete tumour regression but insufficient to achieve long-term local cure was shown to result in accelerated metastasis. A highly significant inhibition of metastasis was observed with the drug ICRF 159, but histological features suggested that its anti-metastatic effect in this system did not depend on morphological changes which might prevent dissemination of tumour cells.**

ALTHOUGH failure to achieve local control of human cancers has been estimated to contribute to the death of about one-third of cancer patients (Suit, 1969), the major cause of failure in cancer therapy is metastatic disease. Furthermore, it is highly likely that if new techniques lead to increased local tumour control, many of the patients concerned will subsequently succumb to metastases. This depressing conclusion is of course due to the establishment of metastases in the pre-clinical phase of tumour growth. A simplified consideration of the life history of a tumour growing exponentially from a single mutant cell indicates that about 26 population doublings are necessary before the tumour reaches a mean diameter of 5 mm (*i.e.* near the limit for clinical diagnosis) while only another 14 doublings are needed to produce a usually lethal tumour mass of 1 kg (Collins, Loeffler and Tivey, 1956). While it is true that the growth rate of many tumours may decelerate when they reach clinical proportions, the example given illustrates the fact that a very large part of the total duration of a neoplasm is subclinical, and it is therefore not surprising that many tumours have already metastasized when first diagnosed. Nonetheless, clinical experience has shown that perhaps the best single prognostic criterion in cancer is the clinical stage or the size of the tumour mass at the time of presentation. One is therefore forced to conclude that the risk of established metastatic disease increases rather dramatically in the clinical or immediate preclinical phase of growth, and cancer therapists must face the fact that they may influence the probability of metastasis by their management. In

* Present address: Department of Experimental Radiotherapy, M.D. Anderson Hospital & Tumor Institute, Houston, Texas 77025, U.S.A.

principle, it should be easy to assess the risks of a particular procedure by reference to controlled animal studies, but in practice no animal tumour system can adequately represent the human problem in every detail.

The most notable objections to the validity of many experimental tumours as models of human cancer are (i) the existence of powerful antigenic differences between tumour and host, often involving transplantation antigens; (ii) patterns of local growth and metastasis (or lack of it), which are often quite unrepresentative of human cancer; (iii) a frequent lack of histological resemblance to the commonly occurring human neoplasms; and (iv) very rapid growth rates. The first 3 of these major objections are overcome in a system which has been developed from a murine squamous carcinoma and the model has been used to investigate some of the factors which have variously been alleged to influence metastasis. In addition, the natural history of the tumour has been studied in relation to its metastatic behaviour (Peters, 1975).

### MATERIALS AND METHODS

*Mice and tumour.*—Female mice of the inbred albino strain WHT/Ht were used throughout these experiments. The tumour, designated Sq. Ca. " G ", arose spontaneously in the forestomach of a female mouse of the WHT/Ht strain in October 1970 and has since been maintained by serial subcutaneous transplantation and by storage in liquid $N_2$. Histologically, the tumour is a squamous cell carcinoma which showed extensive keratinization early in its transplant history, but for the greater part of the duration of the experiments described in this paper (representing passage numbers 53–99) it has been less well differentiated, though readily recognizable as a squamous carcinoma.

Quantitative transplantation assays of the tumour by the subcutaneous route show a $TD_{50}$ (mean number of viable tumour cells required to achieve 50% " takes ") of 200–300 cells when viable cells are injected alone, and 2–7 cells when viable cells are injected in association with an excess of lethally irradiated (LI) cells. The transplantation kinetics of the tumour are consistent with single cell transplantation when excess LI cells are admixed, but are " anomalous " (Porter, Hewitt and Blake, 1973) when viable cells are injected alone, *i.e.* upwards of $10^4$ cells are required for 100% successful transplantation.

In both circumstances, the median latent period between injection of cells and the formation of palpable tumours is inversely correlated with the logarithm of the number of viable cells injected.

In the experiments described in this paper, transplantation was performed intradermally. An inoculum of $10^5$ cells in 0·01 ml produced, on average, discoid tumours of about 120 $mm^3$ in 10 days. However, due in part to variable leakage of the cell suspension from a superficial intradermal injection, a spread of latent periods often occurred. In the experiments involving regional lymph node excision and putative immunization a reduced inoculum of about $2 \times 10^4$ cells was used to produce a longer exposure to tumour growth before a specific size was attained.

Preliminary experiments with the tumour system showed that local excision of primary implants in the first 5 days after inoculation, or when the tumour was no larger than 20 $mm^3$, resulted in no metastases within a 150-day observation period. On the other hand, no mouse in which the primary implant reached a size of 200 $mm^3$ or more, irrespective of its duration of growth, failed to develop metastases. For this reason, and to increase the analogy with human tumours which present as a function of size rather than duration, it was decided to use tumour size as the criterion for randomizing mice into their appropriate experimental group. Subsequent analysis of the data, however, indicated that the correlation of size with metastatic risk was not statistically significant within the range employed (Peters, 1975).

The choice of a tumour size range which resulted in an intermediate risk of metastasis permitted the detection of either increased or decreased risk with different procedures.

The primary inoculation site was on the left flank of the mouse, from which metastasis, when it occurred, was most commonly to the lungs and mediastinum (97% of cases) and to the ipsilateral axillary lymph node (35%). Mice which developed thoracic metastases were prone to sudden death due to erosion of the great vessels, but whenever possible mice were killed when they developed respiratory distress, when a metastatic node reached a size of 1·0 cm diameter, or when other evidence of metastatic disease declared itself. All mice were autopsied to record the extent of metastatic disease and in the course of the experiments involving 280 autopsies, macroscopic metastatic deposits were observed in ovaries (10 cases), liver (6 cases), and kidney, adrenal, brain and bone (1 each). For the purposes of analysis, mice killed for humane reasons in a preterminal state are treated as having " died " on the same day.

Metastases were classified as predominantly thoracic or predominantly lymph nodal, according to which site was directly responsible for death or sacrifice of the mouse. Survivors were observed for at least 150 days and some for up to 12 months. In the whole series of experiments, 280 mice developed metastatic disease: of these, only 2 had a disease-free interval exceeding 100 days, so that survival for 150 days was reasonably regarded as " cure ". No mice died of causes other than metastatic disease.

*Tests for tumour immunogenicity.*—Mice were subjected to live cell " immunization " either by excision of a growing tumour 10 days after implantation (at a size of 30–50 $mm^3$) or by subthreshold intradermal inoculations of live tumour cells. Fourteen days after the initial cell injection, the " immunized " mice and an age-matched group of untreated controls were challenged with subcutaneous injections of viable cells with or without added radiation killed cells. The tumour take incidence and median latent periods for palpable tumour development were recorded.

*Irradiations.*—Local tumour irradiations were performed 4 at a time, using a Westinghouse x-ray machine (250 kVp; 15 mA; 1·3 mm Cu h.v.l.; 494 rad/min). Mice were lightly sedated with tribromethanol (Avertin) 150 mg/kg s.c. and were placed in individual lead boxes which had been specially constructed to allow the flap of skin bearing the tumour to be drawn out from the box onto a perspex stage during the irradiation. The flap was loosely tethered to a central pillar to prevent retraction but care was taken to avoid tension which could have altered blood flow.

*Tumour excisions.*—Mice were anaesthetized with ether and an ellipse of skin including the tumour, with 3–5 mm margins, was excised. The cutaneous site of tumour implantation allowed clean and complete excision, and only 12/342 mice developed local recurrence.

*Axillary lymphadenectomy.*—An arcuate incision was made along the lower border of the pectoralis major and the muscle was lifted forwards to expose the axilla. One large node was regularly present in relation to the axillary vein and in some instances a smaller second node was present. These were removed by teasing free the fatty tissue in which they were embedded. The axillary vein was not removed.

*Incision biopsy.*—A deep wedge of tissue including the full diameter of the tumour plus skin on either side was removed. Topical thrombin was applied if necessary to stop bleeding.

*Tumour manipulation.*—Tumours were gently rolled between finger and thumb for 1 min. This caused softening of the tumour followed by engorgement with blood over the next 10–15 min.

*ICRF* 159.—Supplies of the drug were kindly provided by Dr K. Hellmann of the Imperial Cancer Research Fund. It was given at a dose of 30 mg/kg daily i.p. or s.c. from the time of tumour inoculation till excision or irradiation.

### RESULTS

### *Effect of " immunization " procedures*

The results of attempted tumour immunization are presented in the Table. Mice in which a growing tumour had been excised before challenge showed a slight reduction in tumour takes compared with the controls or with mice which had received previous subthreshold inoculations. None of the differences is statistically significant and no significant prolongation of the tumour latent periods was observed in the " immunized " mice. It is therefore concluded that the tumour is non-immunogenic by these criteria as one would expect for a syngeneically transplanted tumour of spontaneous origin.

TABLE.—*Effect of Prior " Immunization " of Mice on the Subsequent Growth of Tumours from Challenge Inocula. (The Method of " Immunization " was either Excision of a Growing Tumour after 10 days or Subthreshold Intradermal Injections of Live Tumour Cells. Mice were Challenged 14 Days after " Immunization ")*

Series I ($10^4$ viable cells)

| Pretreatment | Tumour incidence | Median latent period |
|---|---|---|
| Nil | 18/20 | 10·5 days |
| Subthreshold intradermal inoculum | 18/20 | 12·0 days |
| Previous tumour growth for 10 days | 17/24 | 12·5 days |

Series II (200 viable cells + $10^5$ lethally-irradiated cells)

| Pretreatment | Tumour incidence | Median latent period |
|---|---|---|
| Nil | 15/20 | 10·5 days |
| Subthreshold intradermal inoculum | 21/24 | 9·5 days |
| Previous tumour growth for 10 days | 18/28 | 10·0 days |

*Effect of pre-operative irradiation*

Four separate experiments involving a total of 145 mice were carried out. In each, tumours were either sham irradiated or given 500 or 2000 rad on reaching a predetermined size which varied from about 30–90 mm³, according to the experiment. The interval between implantation and irradiation varied from 6 to 16 days. All tumours were excised 24 h after irradiation. The combined results are plotted in Fig. 1, where it can be seen that mice receiving 2000 rad pre-operatively had a better survival than either of the other groups. Long-term survival in the 2000 rad group was 20/42 compared with 14/54 in the controls. This difference is statistically significant ($\chi^2 = 4{\cdot}86$; d.f.1; $P < 0{\cdot}05$). Survival in the 500 rad group was 13/49. The patterns of metastatic disease within the groups showed no significant difference, with predominantly thoracic metastases accounting for 72·5% of deaths in the controls, 66·7% in the 500 rad group and 77·3% in the 2000 rad group.

*Effect of regional lymphadenectomy*

Two experiments involving 58 mice were performed. In each, a reduced inoculum was used to permit a longer period of exposure of the regional node to tumour before excision was performed. A size of 90 mm³ was chosen which was reached in 10–29 days after implantation.

FIG. 1.—Effect of pre-operative irradiation with 2000 rad (———) or 500 rad (—·—·—) 24 h before tumour excision on the subsequent development of metastatic disease.

Matched pairs of mice whose tumours reached the reference volume on the same day were allocated randomly to the lymphadenectomy or sham operated control group giving 29 mice in each group. Axillary dissection was done immediately before tumour excision. The results show no significant difference in survival (8/29 dissected and 9/29 controls) and the patterns of metastasis were similar, with predominantly thoracic metastases in 90% of mice dying in the dissected group and 95% of the controls. Curiously, there was a relative deficiency of axillary metastases in the sham operated mice compared with other control groups. This is very likely a chance observation although it is possible that alterations in lymph flow after surgery could be implicated.

*Effect of tumour manipulation*

In 3 experiments involving 33 mice (16 controls, 17 manipulated) 60 or 90 $mm^3$ tumours were used, with growth periods of 8–20 days. Mice were allocated randomly to manipulation or control groups as their tumours reached the reference volume. Manipulation was performed as described in the Materials and Methods section, and excision was performed 15 min later. This time was designed to simulate rough handling of a tumour during operative excision. The results show no significant difference in survival (5/17 manipulated and 4/16 controls). While there was an increased incidence of nodal metastases in the manipulated group (25% c.f. 0%), the difference is not statistically significant ($\chi^2 = 3{\cdot}69$; d.f.1; $P > 0{\cdot}05$) with the numbers of mice available.

*Effect of incisional biopsy*

Two experiments involving 38 mice were performed. Tumours which had been growing for 9–13 days were biopsied as described in the Materials and Methods section at a size of 60 or 90 $mm^3$. Excisions were performed 24 h later on matched pairs of mice (19 per group). The results show no difference in survival between the two groups (5/19 in both) and the difference in metastatic pattern—64% predominantly thoracic in the biopsy group *vs* 78% in the controls—was not significant.

*Effect of non-curative irradiation*

Radiation studies on the tumour system (to be reported elsewhere) indicated that the mean curative dose ($TCD_{50}$) for tumours of about 90 $mm^3$ was around 5000 rad. The fate of mice with tumours in the size range 60–120 $mm^3$ was determined according to whether they received a dose of 4500 rad (*i.e.* low probability of cure) or 5500–6000 rad (high probability of cure). Both doses were sufficient to cause complete regression of tumours and only those mice in which the primary tumour site was macroscopically clear at the time of death were scored for the development of metastases. This analysis, Fig. 2, showed that mice receiving a statistically non-curative dose had a shorter median survival time and poorer long-term survival (0/29) than mice locally "cured" by radiation (9/28) or a third group treated by surgical excision (4/25). The implication is that microscopic recurrence of tumour contributed to the development of distant metastases (see Discussion).

*Effect of ICRF* 159

In the course of experiments performed in collaboration with Dr W. Boggust of St Luke's Hospital, Dublin, to assess the anti-metastatic potential of various chelating agents, a pilot study of the effect of ICRF 159 was carried out which indicated that the drug inhibited metastases in this system. Subsequent experiments confirming this finding are presented here.

Mice were treated with daily injections of 30 mg/kg i.p. or s.c. from the time of tumour implantation until they reached a size of 110–125 $mm^3$ 8–10 days later. Tumours were then either surgically

FIG. 2.—Effect of radiation therapy sufficient to cause complete macroscopic regression of a tumour but adequate to achieve long-term cure (————) compared with locally curative irradiation (——○——) or excision (------) on the subsequent development of metastases.

FIG. 3.—Effect of ICRF 159 30 mg/kg daily i.p. or s.c., from the time of tumour implantation till locally curative treatment on the subsequent development of metastases.

excised or ablated with radiation. Of these mice, 29/40 were long-term survivors compared with 0/8 in a concurrent control group (Fig. 3). The overall survival of control mice from all experiments was 44/179. This is significantly poorer than the survival rate for ICRF 159 treated mice ($\chi^2 = 33{\cdot}8$; d.f.1; $P < 0{\cdot}001$). The growth rate of primary tumour implants was not appreciably affected by the drug; the mean delay in reaching the specified size in the treated group was $<1$ day compared with the controls. Histological examination of tumours from ICRF 159 treated and control mice was made for the features described by Salsbury, Burrage and Hellmann (1970) and James and Salsbury (1974) in the Lewis lung tumour. The degree of haemorrhage at the growing margin of the tumour, the presence of sinusoidal tumour cell-lined blood spaces and the extent of muscle

(a)

(b)

Fig. 4.—Sections from the growing edge of tumours in mice treated with ICRF 159 30 mg/kg/day from the day of tumour cell injection till the day of excision. Panel (a) shows tumour cells lining a sinusoidal blood filled space in the centre-left of the picture. Interstitial haemorrhage is also present. Panel (b) shows active invasion of muscle by the advancing tumour. These appearances do not support the proposition that ICRF 159 acts to prevent access of tumour cells to the circulation. H. and E. ×250.

invasion were assessed. This author was unable to find any consistent differences in these features (Fig. 4), nor was there any difference between the appearances of the primary growths in ICRF 159 treated mice which did or did not develop metastases. Further consideration of this topic is found in the Discussion.

### DISCUSSION

Whether or not a diagnostic or therapeutic manoeuvre influences the probability of metastasis of a particular tumour will depend on the unique relationship between that tumour and its host. Extrapolation of data from one species to another, or even generalization concerning cancer of a particular site or histology in man, can be misleading. Yet to obtain insight into the risks of various procedures necessitates one or other of these approaches. When animal tumour systems are employed, biological variables can be closely controlled and accumulation of data is relatively easy, so that " significant " results are obtainable. But before accepting " significant " results from animal systems it is of paramount importance to ensure that the tumour–host system used is a reasonable model of human cancer. Such a claim can be made for the system used in these experiments.

Two important findings deserve comment in relation to previously reported results: firstly, that regional lymphadenectomy does not adversely affect survival and secondly, that pre-operative radiation is likewise not harmful and may actually be beneficial in terms of survival.

Concerning the role of the regional lymph node, the experimental work of Crile (1965, 1968) is frequently quoted. Of the systems he used, however, one is allogeneic (Sarcoma 180 in non-inbred " Swiss " mice), where H2 locus transplantation antigens were so powerful that 30% of untreated mice rejected the tumour and even then the regional node was of unique significance only in the period 4–10 days after tumour implantation. Clearly such a system cannot be considered a fair model of human cancer. In 2 other systems, nominally isologous but lacking substrain specificity, a direct comparison of radical and simple surgery showed a difference in the incidence of distant metastases in only one and here, again, the time of surgery was critical.

McCredie, Inch and Cowie (1972) found that excision or irradiation of regional lymph nodes 20 days after tumour implantation was without effect on the metastasis of either strongly or weakly antigenic tumours, while Hammond and Rolley (1970), using a syngeneic, chemically induced mouse sarcoma which was weakly immunogenic, could not display any differences in local recurrence or distant metastasis between mice undergoing regional lymphadenectomy and controls. The present experiments, using a spontaneously arising, syngeneically transplanted squamous carcinoma with a range of exposure times, also show no adverse effect of regional lymphadenectomy, leading one to conclude that experimental evidence from representative animal tumours does not support the view that potentially involved regional lymph nodes should be left untreated.

A discussion of the question of preoperative irradiation in relation to the development of metastases has recently been provided by Scott (1972), who identified many of the problems inherent in interpreting clinical and experimental studies. Nickson and Glicksman (1972) lamented the fact that the published experimental data " involve host–tumour reactions which may result from non-isogenic tumours as well as other factors which may be at issue. . . .". The tumour system used in these experiments is free of this criticism as it arose spontaneously and has been maintained in a strictly syngeneic substrain of mice. No deleterious effect of pre-operative irradiation was observed: indeed 2000 rad given 24 h before excision was positively beneficial. These results are in accord with those of Sheldon (1974) who studied a slowly growing syngeneic mouse sarcoma of spontaneous origin, as well as those of several other workers (see Perez, 1970.) Reports of increased metastases attributable to irradiation fall into 2 main groups: (i) situations where the tumours in unirradiated controls have reached such massive sizes as to cause constitutional depletion (*e.g.* Kaplan and Murphy, 1949); (ii) studies involving tumours which infiltrate diffusely rather than grow as stromated tumours (*e.g.* van den Brenk and Sharpington, 1971). The consensus of data from experiments which more closely simulate the majority of human cancer leads to the conclusion that the local benefits of pre-operative irradiation need not be sacrificed for fear of promoting metastases.

Mechanical disturbance of the primary tumour by biopsy or manipulation did not significantly influence metastases in this tumour system. In general, clinical experience supports this view, *e.g.* Griffiths *et al.* (1972) found no correlation between the presence of malignant cells in peripheral or local venous blood during resection of colonic and rectal cancers and the subsequent development of metastases, although histological evidence of venous invasion did carry a worse prognosis. Growth of tumour into veins very likely increases the risk of dissemination of large emboli of tumour cells, which have been shown experimentally to be more efficient than single cells in establishing "metastases" (Thompson, 1974), and it is logically prudent to avoid unnecessary rough handling of all neoplasms. Biopsy of cancers for diagnostic and prognostic purposes is generally accepted as a justifiable procedure although the potential hazard of opening into vessels is recognized. Clinical experience with squamous cell carcinomata at various sites, as well as the present experimental results, suggests that these tumours can be biopsied with impunity. In the case of malignant melanomata, radical excision is usually performed on the basis of a clinical diagnosis, for fear of increasing the risk of metastases. However, the evidence to support such a policy is tenuous: Jones *et al.* (1968) and Epstein, Bragg and Linden (1969) both found no evidence of an adverse prognosis in patients whose malignant melanomata were biopsied or locally excised before more radical treatment.

The effect of non-curative radiation on the development of metastases is most interesting. Suit, Sedlacek and Gillette (1970) and more recently Sheldon *et al.* (1974) reported an increased incidence of metastasis in C3H mice which developed recurrent mammary tumours after irradiation; this incidence was reduced by surgical excision of the recurrent tumour. The former authors also determined, on the basis of transplantation assays, that mice dying of metastasis with no macroscopic recurrence did not have an increased incidence of residual subclinical disease. This conclusion, based on only 11 mice because of low incidence of animals in this category, appears to conflict with the results reported here in which mice whose primary sites were macroscopically "clear" after statistically non-curative irradiation had a worse prognosis than those which were locally "cured" whether by radiation or surgery. However, the squamous cell carcinoma used in these experiments is a highly malignant tumour which may well metastasize successfully at an earlier stage in the growth of a recurrence than the less aggressive C3H mammary tumour. All these data indicate that failure to achieve local control of a primary cancer can increase the risk of metastatic disease and underline the importance of radical local cure in the overall strategy of cancer treatment.

The efficiency with which ICRF 159 inhibited the development of metastases in this tumour system is striking. Histological studies of the Lewis lung tumour by Salsbury *et al.* (1970) have indicated that in mice treated with ICRF 159, the tumours showed less haemorrhagic tendency at the growing margin, and that there was a "normalization" of the abnormal, tumour-cell lined blood vessels seen in that tumour. This observation suggested that the drug might act to prevent access of malignant cells into the circulation. James and Salsbury (1974) also reported inhibition of muscle invasion by tumour in mice treated with ICRF 159 at doses of 30 mg/kg/day. In the tumour system used for these experiments, however, I could detect no *consistent* alteration in any of these features (Fig. 4). This of course does not preclude some modification of the tumour microvasculature by the drug but does suggest that the morphological changes seen in the Lewis lung tumour are not essential for the anti-metastatic effect of ICRF 159. Although ICRF 159 has a cytotoxic effect (Sharpe *et al.*, 1970) it did not significantly delay the growth of primary tumour implants in these experiments. One possibility, that the drug could be significantly more toxic to cells which have entered the circulation than to those in a stromated tumour, was rejected by James and Salsbury (1974) using arguments based on drug concentrations in the blood; but the effectiveness of 5-fluorouracil (Hellman *et al.*, 1973) in preventing metastases from the Lewis lung tumour at a dose which did not retard primary growth suggests a higher susceptibility of disseminated cells to that cytotoxic agent. Moreover, the report of Hellmann, James and Salsbury (1974) that "artificial" lung metastases from intravenously injected Lewis lung tumour cells were inhibited by ICRF 159 seems to provide direct evidence in favour of some action on the tumour cells *after* they have gained access to the circulation. The same authors, however, contend that a toxic effect of ICRF 159 on circulating cells cannot explain the efficacy of treatment during the first 6 days of tumour growth. This contention rests on the tenuous proposition that because no metastases develop in mice having their primary tumour implant excised in the first 5–6 days of growth one can assume that no tumour cells have disseminated during this time. It seems fair to conclude that the mechanism(s) by which ICRF 159 inhibits metastases in different systems must remain, for the present, *sub judice*.

I should like to thank Dr H. B. Hewitt for providing the tumour and mice used in these experiments; Misses E. Blake, S. Ayres and A. Walder for assistance, at different times, with the experiments; and Mr M. Stone for help with the diagrams, histology and photography. This work was performed while the author was supported by the Cancer Research Campaign at the Gray Laboratory, Mount Vernon Hospital, Northwood, Middlesex.

### REFERENCES

Collins, V. P., Loeffler, R. K. & Tivey, H. (1956) Observations on Growth Rates of Human Tumors. *Am. J. Roentg.*, **76**, 988.

Crile, G. (1965) Rationale of Simple Mastectomy without Irradiation for Clinical Stage I Cancer of the Breast. *Surgery, Gynec. Obstet.*, **120**, 975.

Crile, G. (1968) The Effect on Metastasis of Removing or Irradiating Regional Nodes of Mice. *Surgery, Gynec. Obstet.*, **126**, 1270.

Epstein, E., Bragg, K. & Linden, G. (1969) Biopsy and Prognosis of Malignant Melanoma. *J. Am. med. Ass.*, **208**, 1369.

Griffiths, J. D., McKenna, J. A., Rowbotham, H. D., Tsolakidis, P. & Salsbury, A. J. (1972) Carcinoma of the Colon and Rectum: Circulating Malignant Cells and Five-year Survival. *Cancer, N.Y.*, **31**, 226.

Hammond, W. G. & Rolley, R. T. (1970) Retained Regional Lymph Nodes: Effect on Metastases and Recurrence after Tumor Removal. *Cancer, N.Y.*, **25**, 368.

Hellmann, K., Salsbury, A. J., Burrage, K., Leserve, A. W. & James, S. E. (1973) Drug Induced Inhibition of Haematogenously Spread Metastases. In *Chemotherapy of Cancer Dissemination and Metastasis*. Eds. S. Garattini and G. Franchi. New York: Raven Press. p. 355.

Hellmann, K., James, S. E. & Salsbury, A. J. (1974) Analysis of the Antimetastatic Effect of the Antimitotic Agent ICRF 159. *Br. J. Cancer*, **30**, 179.

James, S. E. & Salsbury, A. J. (1974) Effect of (±)-1,2-Bis(3,5-dioxopiperazin-1-yl) propane on Tumor Blood Vessels and its Relationship to the Antimetastatic Effect in the Lewis Lung Carcinoma. *Cancer Res.*, **34**, 839.

Jones, W. M., Jones Williams, W., Roberts, M. M. & Davies, K. (1968) Malignant Melanoma of the Skin: Prognostic Value of Clinical Features and the Role of Treatment in 111 Cases. *Br. J. Cancer*, **22**, 437.

Kaplan, H. S. & Murphy, E. D. (1949) The Effect of Local Roentgen Irradiation on the Biological Behaviour of a Transplantably Mouse Carcinoma. I. Increased Frequency of Pulmonary Metastases. *J. natn. Cancer Inst.*, **9**, 407.

McCREDIE, J. A., INCH, W. R. & COWIE, H. C. (1972) Effect of Excision or Local Radiotherapy to a Tumor and its Regional Nodes on Metastases. *Cancer, N.Y.*, **31**, 983.

NICKSON, J. J. & GLICKSMAN, A. S. (1972) Pre-operative Radiotherapy. In *Modern Trends in Radiotherapy*, 2. Ed. T. J. Deeley. London: Butterworths. p. 123.

PEREZ, C. A. (1970) Pre-operative Irradiation in the Treatment of Cancer: Experimental Observations and Clinical Implications. In *Frontiers of Radiation Therapy and Oncology*, **5**. Ed. J. M. Vaeth. Basel: Karger. p. 1.

PETERS, L. J. (1975) The Natural History of Metastasis of a Syngeneic Murine Squamous Carcinoma and the Prognostic Implications of Primary Tumour Size and Duration of Growth. *Br. J. Cancer*, **32**, 366.

PORTER, E. H., HEWITT, H. B. & BLAKE, E. R. (1973) The Transplantation Kinetics of Tumour Cells. *Br. J. Cancer*, **27**, 55.

SALSBURY, A. J., BURRAGE, K. & HELLMANN, K. (1970) Inhibition of Metastatic Spread by ICRF 159: Selective Deletion of a Malignant Characteristic. *Br. med. J.*, iv, 344.

SCOTT, O. (1972) Pre-operative and Post-operative Irradiation. *Br. med. Bull.*, **29**, 59.

SHARPE, H. B., FIELD, E. O. & HELLMANN, K. (1970) Mode of Action of the Cytostatic Agent "ICRF 159". *Nature*, **226**, 524.

SHELDON, P. W. (1974) The Effect of Irradiating a Transplanted Solid Sarcoma on the Subsequent Development of Metastases. *Br. J. Cancer*, **30**, 416.

SHELDON, P. W., BEGG, A. C., FOWLER, J. F. & LANSLEY, I. F. (1974) The Incidence of Lung Metastases in C3H Mice after Treatment of Implanted Solid Tumours with X-rays or Surgery. *Br. J. Cancer*, **30**, 342.

SUIT, H. D. (1969) Introduction. Statement of the Problem Pertaining to the Effect of Dose Fractionation and Total Treatment Time on Response of Tissue to X-irradiation. In *Time and Dose Relationships in Radiation Biology as Applied to Radiotherapy*. NCI-AEC Conf. Proc., Carmel, U.S.A. p. 5.

SUIT, H. D., SEDLACEK, R. S. & GILLETTE, E. L. (1970) Examination for a Correlation between Probabilities of Development of Distant Metastasis and of Local Recurrence. *Radiology*, **95**, 189.

THOMPSON, S. C. (1974) The Colony Forming Efficiency of Single Cells and Cell Aggregates from a Spontaneous Mouse Mammary Tumour using the Lung Colony Assay. *Br. J. Cancer*, **30**, 332.

VAN DEN BRENK, H. A. S. & SHARPINGTON, C. (1971) Effect of Local x-irradiation of a Primary Sarcoma in the Rat on Dissemination and Growth of Metastases: Dose–Response Characteristics. *Br. J. Cancer*, **25**, 812.